# A comparative in silico analysis of the vlhA gene regions of Mycoplasma gallisepticum and Mycoplasma synoviae isolates from commercial hen farms in Mexico

**DOI:** 10.1099/acmi.0.000760.v4

**Published:** 2025-02-21

**Authors:** Linda M. Maya-Rodríguez, Gabriela Gómez-Verduzco, Francisco J. Trigo-Tavera, Leticia Moreno-Fierros, Verónica Rojas-Trejo, Rosa Elena Miranda-Morales

**Affiliations:** 1 Departamento de Microbiología e Inmunología, Facultad de Medicina Veterinaria y Zootecnia, Ciudad Universitaria, Universidad Nacional Autónoma de México, CDMX, 04510, Mexico; 2 Departamento de Medicina y Zootecnia de Aves, Facultad de Medicina Veterinaria y Zootecnia, Ciudad Universitaria, Universidad Nacional Autónoma de México, CDMX, 04510, Mexico; 3 Departamento de Patología, Facultad de Medicina Veterinaria y Zootecnia, Ciudad Universitaria, Universidad Nacional Autónoma de México, CDMX, 04510, Mexico; 4 Facultad de Estudios Superiores Iztacala, Unidad de Biomedicina (UBIMED), Los Reyes Ixtacala, Universidad Nacional Autónoma de México, Tlanepantla de Baz, 54090, Mexico

**Keywords:** avian, lipoprotein, *Mycoplasma*

## Abstract

Avian mycoplasmosis, caused by *Mycoplasma synoviae* and *Mycoplasma gallisepticum*, poses significant economic challenges due to respiratory issues, reduced egg production and soft eggshells. The variable lipoprotein haemagglutinin (VlhA) protein, crucial for pathogenicity, comprises conserved (MSPB) and variable (MSPA) regions. The aim of this study was to identify the conserved region of *vlhA* gene sequences in field strain. We examined *vlhA* sequences from field strains collected in central Mexico (Jalisco and Mexico City). Specifically, we analysed 124 deformed eggs and 10 laying hens from 9 farms with Hy-line and Bovans breeds. Using PCR targeting the *mgc2* and 16S rRNA genes, we characterized 24 field strains, 4 of which were *Myc. synoviae* and 20 of which were *Myc. gallisepticum*. We analysed the *vlhA* regions, based on the AF035624.1 reference sequence, with American Type Culture Collection strains as positive controls. Additionally, we validated the PCR with 20 negative samples from *Mycoplasma* isolation without the need for cultivation. We identified two amplification regions: MSPB and MSPA. Bioanalysis revealed relationships between our field samples and avian *Mycoplasma* sequences in GenBank, alongside similarities with lipoproteins present in *Acholeplasma laidlawii* PG8 and *Escherichia coli*. Given the significance of the VlhA protein in pathogenicity and immune evasion, the identified conserved sequences hold potential as therapeutic targets and for phylogenetic studies.

## Data summary

We used the following sequences reported in GenBank to build phylogenetic trees: 16S rRNA from *Mycoplasma synoviae* (NZ_CP021129.1 and AY623915.1); *vlhA* from *Myc. synoviae* (AF035624.1, KC506819.1, KC506816.1, JX233549.1, JX233548.1, JX233547.1, KR232818.1, KR232811.1, KR232804.1, KR232803.1, KJ722788.1, KJ722787.1, KC506822.1, KC506820.1, KC506819.1, KC506816.1, JX233549.1 and KU577585.1); *mgc2* from *Mycoplasma gallisepticum* (JQ770176.1, KJ019177.1, MZ079374.1, KX268633.1, KP300758.1, KP279742.1 and HQ143373.1); and 16S rRNA from *Myc. gallisepticum* (KP704286.1:c645791-645249 and CP044225.1).

**Impact statement**

Variable lipoprotein haemagglutinin (VlhA) regulates the immune system, stimulates inflammation and promotes immune evasion. This surface protein acts as an adhesin by binding to receptors containing sialic acid in cell–epithelium glycoconjugates, making it important in pathogenicity and a vaccine candidate. The purpose of this study was to identify two homologous regions in the sequences of the *vlhA* gene common in Mexican field strains, using ATCC strains as controls. The amplified regions were also present in *Acholeplasma laidlawii* PG8 and *Escherichia coli*. The bioinformatic analysis showed similar nucleotide sequences, so we presume that there are ancestral sequences. Nevertheless, additional studies are needed to confirm this hypothesis.

## Introduction

Mycoplasmosis is an infectious disease in birds that causes soft shells and respiratory problems. The most frequently associated species with this disease are *Mycoplasma gallisepticum* and *Mycoplasma synoviae* [1,2], both of which are transmitted horizontally and vertically [3,4]. In laying hens, these species infect the respiratory tract, spreading into the air sacs, left ovary and oviduct, resulting in decreased egg laying and reduced eggshell quality [5]. Egg production can decrease by 10–20% in birds infected by *Myc. gallisepticum* and by 5–10% in birds infected by *Myc. synoviae* [4,6, 7]. Some authors have demonstrated the presence of *Myc. synoviae* in hens experimentally infected with a *Myc. synoviae* type strain (WVU1853), establishing the possibility of isolating *Mycoplasma* from deformed eggs [4].

The diagnosis of *Myc. gallisepticum* involves PCR using the *mgc2* gene [8], an adhesin protein, whereas for the diagnosis of *Myc. synoviae*, the variable lipoprotein haemagglutinin (*vlhA*) gene is used [5]. Noormohammadi *et al*. [9] reported the *vlhA* sequence in GenBank under AF035624.1; it is 2405 bp. The two primers used in the Organización Mundial de la Salud Animal OMSA/Word Organisation for Animal Health WOAH [5] were reported by Bencina *et al.* [10,11]; they are from the same region amplified in two *Myc. synoviae* type strains (K1968 and WVU1853) and are used for variability studies. When studying the amplification of primers in different strains, Wetzel *et al.* [12] and El-Gazzar *et al.* [13] observed that not all primers could amplify a product in all field strains. In the diagnosis of *Myc. synoviae*, the inhibition of haemagglutination for haemadsorption by the VlhA protein is common because this protein has a binding motif for sialic acid, which covers red blood cells [14,15].

In mycoplasmosis, lipoproteins play an important part in the infection and evolution of the bacteria. *Mgc2* is a lipoprotein important for colonization [16]. VlhA is a major surface lipoprotein present on *Myc. synoviae* and *Myc. gallisepticum*; this protein is cleaved into two regions: MSPB and MSPA. The MSPB region is genetically more stable and associated with the cell membrane in the N-terminal region [11]. In contrast, the MSPA region, located near the C-terminus, is considered to be variable because it has a greater recombinant capacity, with paralog genes that flank the coding gene. VlhA has haemagglutinin capacity, and it is antigenic and responsible for stimulation and evasion of the immune system [9,13]. Both MSPA and MSPB bind to sialic acid receptors linked with glycoprotein on the surface of epithelial cells [11]. VlhA is a major antigenic component with 23 other proteins and a target diagnostic candidate [17,18]. The *Myc. synoviae* MS53 strain is structured by 64 genes, of which the *vlhA* gene accounts for 8% of the entire genome [13]. In contrast, *Myc. gallisepticum* has only 43 genes related to pathogenicity [19].

VlhA plays an important biological, pathogenic and virulent role in bacteria [20]. A previous study discussed its relevance in the survival of bacteria because of the capacity of recombination with paralog genes and gene transfer between *Myc. synoviae* and *Myc. gallisepticum* [21]. Given the importance of VlhA for the detection of mycoplasmosis, the aim of this study was to obtain sequences of *vlhA* – specifically, the MSPA and MSPB regions – from *Myc. synoviae* and *Myc. gallisepticum* field strains isolated in central Mexico and to compare them with reported sequences of strains from India, Brazil and Europe, as well as with vaccine strain sequences stored in GenBank.

## Methods

### Study area and samples

In Mexico, it is a challenge to obtain samples to study mycoplasmosis in laying hens due to the strict biosecurity guidelines implemented in commercial poultry farms. Therefore, this study utilized 124 deformed eggs from hens suspected of having mycoplasmosis, collected from 9 commercial layer farms housing Hy-line and Bovans White lineage birds. Additionally, at one of these farms, ten hens at the end of their production cycle were collected and processed according to the criteria outlined by the Subcommittee for the Care and Use of Experimental Animals (SICUAE), following the approved protocol in the SICUAE.DOC-2020/1–1 document. In total, ten oviduct samples and ten air sac samples were processed. All samples are considered disposable in commercial hen farms. These farms are situated in the central region of Mexico (Mexico City and Jalisco), and the laying hens were not vaccinated against *Myc. synoviae* and *Myc. gallisepticum*. The production of deformed eggs in laying chickens can be a consequence of mycoplasmosis, thereby increasing the likelihood of isolating *Mycoplasma* strains [4,22].

### *Mycoplasma* isolation from samples

*Mycoplasma* was isolated following the methodology of Kleven and Bradbury [5], using Frey’s culture medium [23]. Each egg was analysed based on its components: egg white, yolk and eggshell membrane. The mucosa of the oviduct (ampulla and isthmus) and left abdominal air sac samples were collected with a sterile swab to isolate *Mycoplasma*. The cultures were incubated in an incubator (Thermo Fisher Scientific, Marietta, OH, USA) for up to 30 days to visualize *Mycoplasma*-like colonies. Then, *Mycoplasma* was identified based on colony morphology, filtering through 0.45 µm filters, and digitonin tests (Biotechnology, Santa Cruz, CA, USA) [23].

*Mycoplasma* isolates were grown to a final volume of 100 ml in Frey’s culture medium. The cultures were seeded on blood agar and Frey’s solid medium to ensure the purity and viability of the strains each time the cultures were spiked with fresh Frey medium, thus favouring *Mycoplasma* growth.

### Testing for the presence of VlhA in the isolated strains

The presence of VlhA in the strains was evaluated based on a qualitative haemadsorption assay and a quantitative haemagglutination assay.

For the haemadsorption assay, all typed field strains (*Myc. synoviae* and *Myc. gallisepticum*) were seeded in Frey’s semisolid medium and incubated for 7 days at 37 °C under microaerobiosis to obtain visible colonies in the solid medium. Each culture was covered with a 0.5% erythrocyte suspension in PBS (pH 7.2) (FMVZ-UNAM) (Facultad de Medicina Veterinaria y Zootecnia-Universidad Nacional Autónoma de México, Mexico City, Mexico) and incubated for 1 h at 37 °C. Finally, the cultures covered with the suspension were washed twice with sterile PBS (pH 7.2) and visualized under an optical microscope (with a 40×objective lens) to determine the adsorption of erythrocytes to the *Mycoplasma* colonies [24,25].

For the haemagglutination assay, 25 µl of PBS (pH 7.2) was deposited in the nine wells of each row of a ‘U’ bottom plate (Ref. 3788, Corning, NY, USA). All strains were adjusted to a bacterial concentration of 3.1×10^6^ UCC (colour change units). Subsequently, of 50 µl of each strain, 25 µl was placed in the first well for 1 : 1 serial dilutions in the following rows. During dilution, 25 µl of erythrocytes at a concentration of 0.5% in PBS was placed in each well. Finally, the plates were incubated for 1 h at room temperature (~20 °C) before reading; to get to haemagglutinin, the title was to take just the haemagglutination in each well [24,25].

### Extraction of genetic material from the *Mycoplasma* isolates for characterization

All typed field strains and American Type Culture Collection (ATCC) strains were centrifuged at 11 300 *g* for 45 min at 4 °C (2007, Thermo Electron Corporation, Karlsruhe, Germany) and washed three times with PBS (pH 7.2). DNA was extracted using guanidine thiocyanate (Mca BioBasic, Amherst, NY, USA) following the methodology described by Sambrook and Russell [26]. This DNA was used for molecular typing with endpoint PCR and MyTaq polymerase (BIO-2110X, Bioline, Cincinnati, OH, USA).

### Molecular typing of *Mycoplasma* isolates by endpoint PCR

The *Myc. gallisepticum* typing was performed by amplifying a fragment encoding the *mgc2* gene using the forward primer 5′-CGCAATTTGGTCCTAATCCCCAACA-3′ (designed with the online IDT tool at https://www.idtdna.com/pages) and the reverse primer 5′-TAAACCCACCTCCAGCTTTATTTCC-3′ [27], with the following thermocycler programme using a Techne 3Prime Personal Thermal Cyclers (Staffordshire, UK): denaturation at 95 °C for 4 min; 30 cycles of denaturation at 94 °C for 30 s, annealing at 55 °C for 1 min and extension at 72 °C for 1 min; and final elongation at 72 °C for 5 min. The amplicon was 349 bp.

For *Myc. synoviae*, the 16S rRNA gene was amplified based on the NZ_CP021129.1 sequence from GenBank. The primers were FW 5′-CAGTCGTCTCCGAAGTTAACAA-3′ and RV 5′-CACAAGCGGTGGAGCATG-3′, under the following thermocycler programme: denaturation at 95 °C for 4 min; 36 cycles of denaturation at 94 °C for 30 s, annealing at 57 °C for 1 min and elongation at 72 °C for 1 min (36 cycles); and final elongation at 72 °C for 5 min. The amplicon was 514 bp.

### Identification and amplification of the MSPB and MSPA regions of *vlhA*

Six internal primers were designed based on the AF035624.1 sequence of GenBank, reported by Noormohammadi *et al.* [9]. For example, the first pair of primers was designed to amplify the MSPB region from nucleotides 77 to 384 of the sequences, producing a 307 bp amplicon (Table 1). The thermocycler programme was denaturation at 95 °C for 4 min; 36 cycles of denaturation at 94 °C for 30 s, annealing at 55 °C for 1 min and elongation at 72 °C for 1 min; and a final elongation at 72 °C for 5 min. The other five sets of internal primers correspond to the transition zone (MSPB–MSPA) and the MSPA region of *vlhA* (Table 1). The same thermocycler programme described above was used for amplification.

**Table 1.** Primers used to amplify the MSPB and MSPA regions of *vlhA* (based on the AF035624.1 reference sequence in GenBank)

Primer name	Sequence (5′→3′)	Temp. (°C)	Amplicon size (bp)
FW MSPB	GCT CCT GCT GTT ATA GCA	55	307
RV MSPB	CTC TGG CTT CAG CTT CTG	55	
FW1 MSPA	CAG AAG CTG A AG CCA GAG	53	374
RV1 MSPA	TAA AGC TGT ATT AAC AAG AGA TTC AAG	53	
FW2 MSPA	CTT GAA TCT CTT GTT AA T ACA GCT TTA	53	397
RV2 MSPA	GAG TAG CTG CTT GAG TTG G	53	
FW3 MSPA	CCA ACT CAA GCA GCT ACT C	53	321
RV3 MSPA	GTC GTA ACC ATC TGC TAC G	53	
FW4 MSPA	CGT AGC AGA TGG TTA CG A C	53	446
RV4 MSPA	GTC GTA ACC ATC TGC TAC G	53	
FW5 MSPA	GCT CAA ACT ATT AAA GAT GTT AAC G	53	542
RV5 MSPA	GCA GTT CCT TGT TGT TGA GTA TCG	53	

### Controls for standardization

The *Myc. gallisepticum* S6 (ATCC No. 15302) and *Myc. synoviae* WUV1853 (ATCC No. 25204) type strains were employed as positive controls and to standardize the molecular tests. These types of strains were processed under the same conditions as the isolates, following the methodology described above. The negative controls were *Staphylococcus aureus* ATCC 6538 and *Escherichia coli* CVET0058 – isolated from a canine urinary sample in 2022 (at the FMVZ-UNAM Diagnostic Laboratory) and typed based on conventional biochemical methods (following the University of California, Davis’s guide under the ISO 9001 standard) – *Acholepasma laidlawii* PG8 NCTC 10116 and Ambion nuclease-free water (AM9916, Thermo Fisher Scientific). These bacterial strains were selected because they contain lipoprotein, but, unlike VlhA, their lipoprotein has not been reported to have a sialic acid binding motif.

All PCR standardizations were based on previous protocols [10,12, 13] and the alienation temperature was adequate. The extracted DNA concentration was determined based on serial dilutions measured with a NanoDrop Lite (Thermo Scientific, Wilmington, DE, USA). All PCR was performed in a 25 µl reaction that contained 7 µl of water, 5.5 µl of buffer, 1 µl of the forward primer, 1 µl of the reverse primer, 10 µl of DNA and 0.5 µl of MyTaq polymerase (Bioline).

### Comparative evaluation of the processing method

A comparative evaluation of the processing method was performed by randomly selecting ten samples that tested negative for digitonin (163C, 74C, 58C, 122C, 103F, B1U, 128Y, 164Y, 163Y and B3M) and ten samples that were negative for cultivation (18F, 76F, 86F, 97F, 98F, 100F, 113F, 126F, 107F and 133F). These 20 samples were centrifuged to concentrate them, and DNA was extracted using guanidine thiocyanate, followed by PCR.

All PCR products were separated by electrophoresis at 75 V for 55 min on a 1.5% agarose gel stained with 0.5 mg ml^−1^ ethidium bromide. The DNA bands were visualized using a Gel Logic 212 Pro photo documenter (Carestream, Woodbridge, CT, USA). The 1 kb HyperLadder, Bioline (SKU: BIO-33025) or 50 bp DNA ladder, Invitrogen (Thermo Fisher Scientific. No. 10416014) was also run on each gel. The PCR products that showed the correct size were sent for purification and sequencing at the Biology Institute of UNAM (Mexico City, Mexico). The sequences were used for bioinformatic analysis using BioEdit and mega X 10.1.6 to determine homology between the field and type strains [1,28,30], with the nucleotide sequences translated based on the *Mycoplasma* genetic code. The *Myc. synoviae* WUV1853 strain sequence was sequenced on two different occasions.

## Results

### *Myc. synoviae* and *Myc. gallisepticum* field strains

Of the 124 egg samples and 10 chickens tested, we identified 24 *Mycoplasma* strains (19.4%). Among these, we recovered 22 (14.5%) from deformed eggs, 4 from egg whites, 7 from yolks and 11 from eggshell membranes. Additionally, there was one strain from an oviduct and one strain from an air sac, for a total of ten studied (10%). These isolates corresponded to 90% of the total number of isolates from eggs and 10% from the oviduct and air sac, respectively.

PCR typing of the obtained strains resulted in 20 *Myc. gallisepticum* strains – 18 from the eggs and 2 from the chickens – 4 *Myc. synoviae* strains (Table 2). These strains came from seven of the nine farms analysed, with three farms having strains from both species. The most frequent egg components for isolation were the eggshell membrane, the yolk and the egg white. On two occasions, we isolated two strains from the same egg (12C, 12Y, 41F, 41Y, 132Y and 132F).

**Table 2.** Results of the haemoadsorption and haemagglutinin assays

Strain identification	Haemoadsorption assay result	Haemagglutination titre	Sample	Species
F3	+	1/32	Shell membrane	*Myc. gallisepticum*
C12	+	1/2	White	*Myc. gallisepticum*
Y12	+	1/2	Yolk	*Myc. gallisepticum*
Y32	+	1/4	Yolk	*Myc. gallisepticum*
F40	+	1/4	Shell membrane	*Myc. gallisepticum*
Y41	+	1/2	Yolk	*Myc. gallisepticum*
F41	+	1/2	Shell membrane	*Myc. gallisepticum*
F42	+	1/2	Shell membrane	*Myc. gallisepticum*
F62	+	1/4	Shell membrane	*Myc. gallisepticum*
C91	+	1/4	White	*Myc. gallisepticum*
F132	+	1/2	Shell membrane	*Myc. gallisepticum*
Y132	+	1/2	Yolk	*Myc. gallisepticum*
C157	+	1/2	White	*Myc. gallisepticum*
F162	+	1/8	Shell membrane	*Myc. gallisepticum*
F231	+	1/2	Shell membrane	*Myc. gallisepticum*
C251	+	1/8	White	*Myc. gallisepticum*
F281	+	1/2	Shell membrane	*Myc. gallisepticum*
Y252	+	1/4	Yolk	*Myc. gallisepticum*
B2M	+	1/2	Oviduct	*Myc. gallisepticum*
B4SA	+	1/4	Air sacs	*Myc. gallisepticum*
Type strain (S6)	+	1/16	ATCC	*Myc. gallisepticum*
Y277	+	1/4	Yolk	*Myc. synoviae*
Y192	+	1/64	Yolk	*Myc. synoviae*
F63	+	1/16	Shell membrane	*Myc. synoviae*
F32	+	1/4	Shell membrane	*Myc. synoviae*
Type strain (WVU1853)	+	1/16	ATCC	*Myc. synoviae*

+ positive haemoadsorption test. F: egg shell membrane, Y: yolk, C: egg white, SA: air sac, M: oviduct.

### Haemoadsorption and haemagglutination assays

We evaluated the presence of VlhA in the *Mycoplasma* strains based on haemoadsorption (qualitative) and haemagglutination (quantitative) assays. Table 2 shows the haemagglutination titres in the strains. The control strains (*Myc. synoviae* WUV1853 and *Myc. gallisepticum* S6) displayed a titre of 1/16, and almost all field strains presented a titre of 1/2 to 1/16; the exceptions were *Myc. gallisepticum* F3 (1/32) and *Myc. synoviae* Y194 (1/64).

### Amplification and analysis of the MSPB and MSPA regions in the analysed strains

**Fig. 1.**
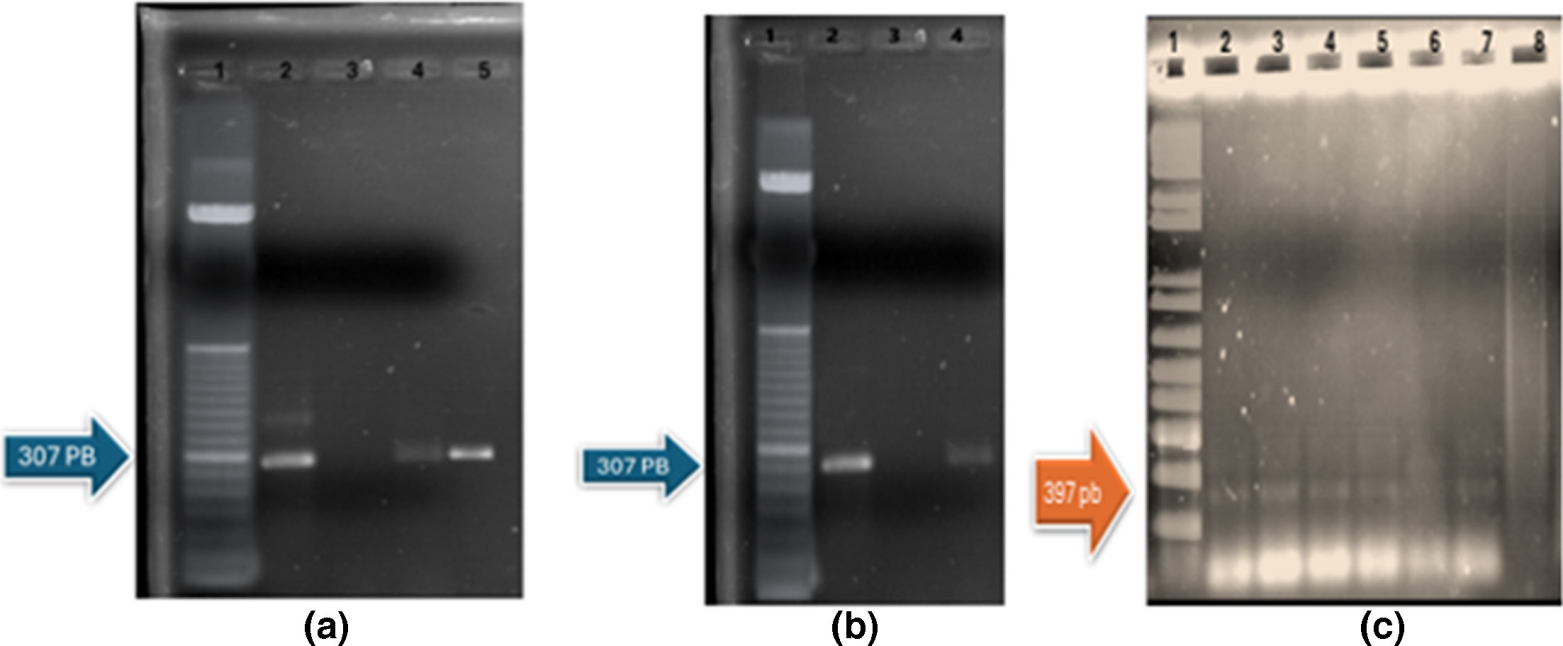
(**a**) A representative agarose gel showing PCR amplification of the 307 bp MSPB region. Lane identities: *Myc. synoviae* (lane 2), Ambion nuclease-free water (lane 3), *E. coli* CVET0058 (lane 4) and *Myc. bovis* (lane 5). (**b**) A representative agarose gel showing PCR amplification of the 307 bp MSPB region. Lane identities: *Myc. synoviae* (lane 2), *S. aureus* ATCC 6538 (lane 3) and *A. laidlawii* PG8 (lane 4). (**c**) A representative agarose gel showing PCR amplification of the 397 bp MSPA fragment 2. Lane identities: *Myc. synoviae* (lane 2), *Myc. gallisepticum* (lane 3), *A. laidlawii* PG8 (lane 4), *E. coli* CVET0058 (lane 5), Ambion nuclease-free water (lane 6), *Myc. bovis* (lane 7) and *S. aureus* ATCC 6538 (lane 8). For each gel, lane 1 contains marker 50 bp ladder (**a, b**) and 1 kb DNA HyperLadder.

We amplified the MSPB region (based on a PCR product of 307 bp) and MSPA fragment 2 (397 bp) in the 24 identified strains (20 *Myc. gallisepticum* and 4 *Myc. synoviae*) as well as the positive control strains *Myc. synoviae* WUV1853 and *Myc. gallisepticum* S6 (Fig. 1). The minimum amount of DNA required for PCR in a final reaction volume of 25 µl was 3 ng µl^−1^. The negative controls, *A. laidlawii* and *E. coli*, also showed the expected amplicons (Fig. 1); the sequences are added in FASTA (available in the online Supplementary Material). Each negative control was run three times and showed the same result each time.

### Sequence analysis of the amplified region of VlhA

After translating the amplified *vlhA* sequences from the *Myc. synoviae* and *Myc. gallisepticum* field strains into the protein sequences, we identified various mutations, including transversion mutations such as alanine (GCT) to proline (CCT), alanine (GCT) to serine (TCT) and threonine (ACA) to arginine (AGA), and one transition mutation from lysine (AAA) to arginine (AGA). These mutations were primarily located in the MSPB region, indicating sequence variability (Table 3). The five internal primers used in the MSPA region only showed amplification of fragment 2, resulting in a 397 bp product in all 24 identified field strains. We found transversion mutations – including a stop codon (TAA) changed to an isoleucine codon (ATT), lysine (AAA) changed to leucine (TTA), lysine (AAA) changed to asparagine (AAT) and alanine (GCA) changed to alanine (GCT) – but they did not interfere with the amplification results (Table 3).

**Table 3.** The number and type of mutations in studied sequences

Sequence	No. of mutations	Mutation type	Total no.
MSPB	32	Transversion	33
MSPB	1	Transition	
MSPA fragment 2	14	Transversion	29
MSPA fragment 2	15	Transition	

**Fig. 2.**
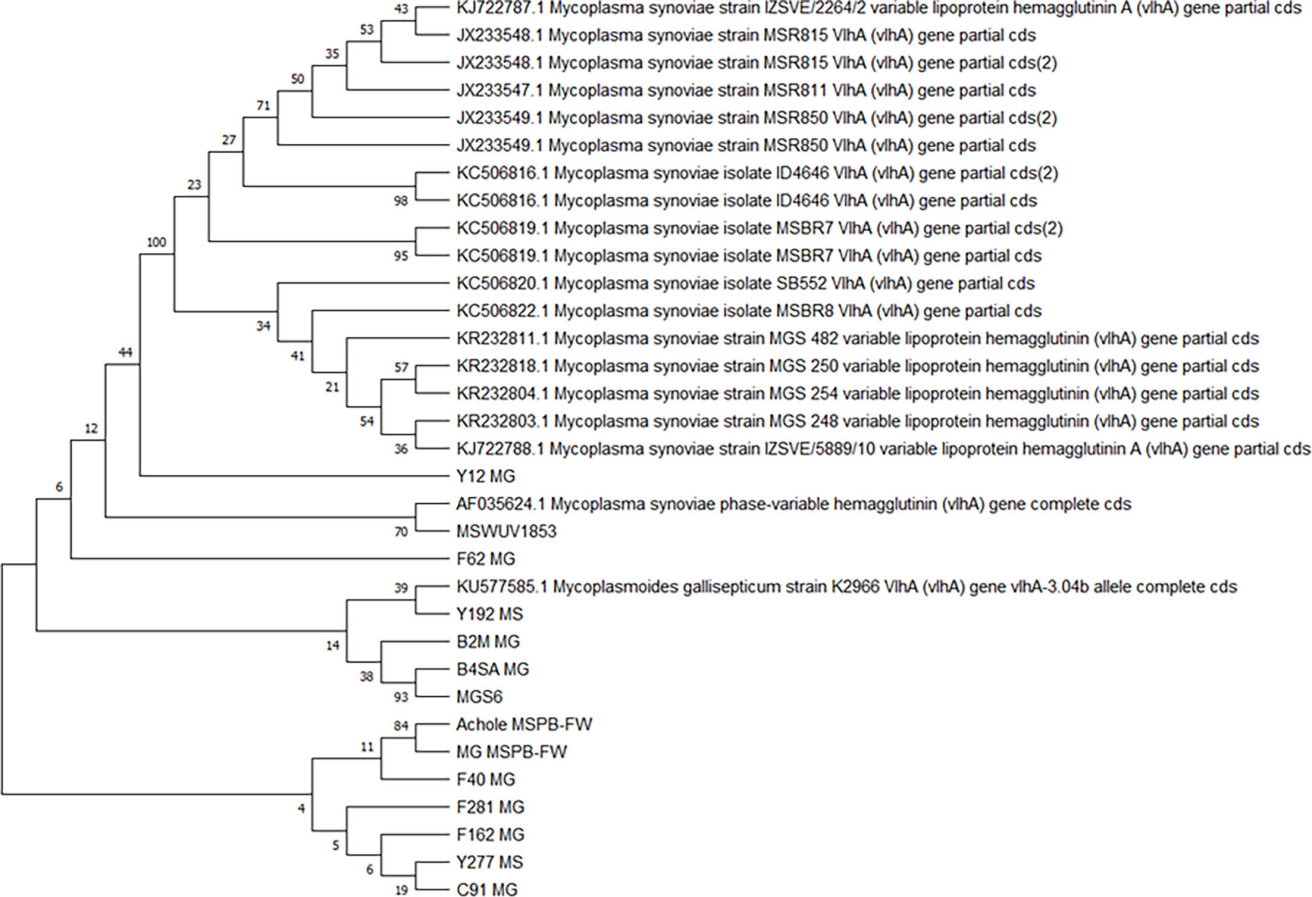
Analysis of the amplified sequences of the MSPB region of *vlhA* (the amplicon was 397 bp). Branches corresponding to partitions reproduced in <50% of the bootstrap replicates are collapsed. The analysis involved 33 nucleotide sequences from 10 field strains (8 *Myc. gallisepticum* [MG] and 2 *Myc. synoviae* [MS]). The reference sequences were *Myc. gallisepticum* S6 (MGS6), *Myc. synoviae* WUV1853 and *Myc. gallisepticum* MSPB-FW (AF035624.1). Additionally, *A. laidlawii* and three positive samples were included, along with 19 sequences retrieved from GenBank. The final dataset comprised a total of 2249 positions. Evolutionary analyses were conducted using mega X. The GeneBank access for sequences is in Table 4.

We analysed all amplified sequences using the blast platform and identified them as *vlhA*. Phylogenetic analysis revealed a relationship with *Myc. synoviae* sequences from other continents (Fig. 2). We also determined the neighbour-joining evolutionary distances via the maximum likelihood method in the mega X software to assess the evolutionary history of *vlhA*. The Mexican field strains were related to strains isolated in Europe and the *Myc. gallisepticum* K2966 strain, associated with the ts-11 vaccine strains, isolated in the USA (KU577585). The *A. laidlawii* sequences showed a relationship with the identified *Myc. gallisepticum* and *Myc. synoviae* field strains and the Mexican strains were linked with other sequences stored in GenBank from the USA, India, Italy, Brazil and Iran.

**Table 4.** The GenBank sequences used to construct the phylogenetic trees

Key sequence	Origin
AF035624.1 *Mycoplasma synoviae* phase-variable haemagglutinin (*vlhA*) gene complete cds	ATCC
KC506819.1 *Mycoplasma synoviae* isolate MSBR7 VlhA (*vlhA*) gene partial cds(2)	Brazil
KC506816.1 *Mycoplasma synoviae* isolate ID4646 VlhA (*vlhA*) gene partial cds(2)	Brazil
KC506822.1 *Mycoplasma synoviae* isolate MSBR8 VlhA (*vlhA*) gene partial cds	Brazil
KC506820.1 *Mycoplasma synoviae* isolate SB552 VlhA (*vlhA*) gene partial cds	Brazil
KC506819.1 *Mycoplasma synoviae* isolate MSBR7 VlhA (*vlhA*) gene partial cds	Brazil
KC506816.1 *Mycoplasma synoviae* isolate ID4646 VlhA (*vlhA*) gene partial cds	Brazil
KC506816.1 *Mycoplasma synoviae* isolate ID4646 VlhA (*vlhA*) gene partial cds	Brazil
KR232818.1 *Mycoplasma synoviae* strain MGS 250 variable lipoprotein haemagglutinin (*vlhA*) gene partial cds	India
KR232811.1 *Mycoplasma synoviae* strain MGS 482 variable lipoprotein haemagglutinin (*vlhA*) gene partial cds	India
KR232804.1 *Mycoplasma synoviae* strain MGS 254 variable lipoprotein haemagglutinin (*vlhA*) gene partial cds	India
KR232803.1 *Mycoplasma synoviae* strain MGS 248 variable lipoprotein haemagglutinin (*vlhA*) gene partial cds	India
JX233549.1 *Mycoplasma synoviae* strain MSR850 VlhA (*vlhA*) gene partial cds(2)	Iran
JX233548.1 *Mycoplasma synoviae* strain MSR815 VlhA (*vlhA*) gene partial cds(2)	Iran
JX233547.1 *Mycoplasma synoviae *strain MSR811 VlhA (*vlhA*) gene partial cds	Iran
JX233548.1 *Mycoplasma synoviae* strain MSR815 VlhA (*vlhA*) gene partial cds	Iran
KJ722788.1 *Mycoplasma synoviae* strain IZSVE/5889/10 variable lipoprotein haemagglutinin A (*vlhA*) gene partial cds	Italy
KJ722787.1 *Mycoplasma synoviae* strain IZSVE/2264/2 variable lipoprotein haemagglutinin A (*vlhA*) gene partial cds	Italy
KU577585.1 Mycoplasmoides gallisepticum strain K2966 VlhA (*vlhA*) gene vlhA-3.04b allele complete cds	USA
JQ770176.1 *Mycoplasma gallisepticum* str. F Mgc2 (*mgc2*) gene partial cds	Australia
KJ019177.1 *Mycoplasma gallisepticum* isolate 2013/UFMG2 cytoadhesin (*mgc2*) gene partial cds	Brazil
MZ079374.1 UNVERIFIED: Mycoplasmoides gallisepticum isolate MGH01 cytadhesin-like (*mgc2*) gene partial sequence	India
KX268633.1 Mycoplasmoides gallisepticum isolate F/2015 MGC2 (*mgc2*) gene partial cds	Thailand
KP300758.1 *Mycoplasma gallisepticum* strain MGS1167 cytadhesin (*mgc2*) gene partial cds	India
KP279742.1 *Mycoplasma gallisepticum* strain MGS 9B cytadhesin protein (*mgc2*) gene partial cds	India
HQ143373.1 *Mycoplasma gallisepticum* strain EGY/75651/CK09 MGC2 gene partial cds	USA
AY623915.1 Mycoplasma synoviae strain WVU1853 16S rRNA gene, partial sequence	Korea

### Comparative evaluation of the processing method

Of the 20 samples, 8 were positive for the MSPB region based on PCR. Among these, six were negative for the digitonin test (163C, 74C, 58C, 122C, 164Y and 163Y), and two were negative for *Mycoplasma* culture (126F and 133F), but both amplified the *mgc2* from *Myc. gallisepticum* with PCR. Based on PCR, the digitonin-negative strains were not *Myc. synoviae* or *Myc. gallisepticum*.

## Discussion

The number of *Mycoplasma* strains we isolated and typed in this work from poultry farms in Mexico aligns with findings from previous studies conducted in France and India, which reported a prevalence of 7–23% [30,31]. The isolation of *Mycoplasma* strains is not necessary for PCR, given that in our comparative evaluation of the processing method, we observed amplification of PCR products without isolation. For endpoint PCR, a high number of bacteria is necessary for amplification, so this test is sensitive but not specific.

Among the samples we collected for this study, we identified a higher frequency of strains in the eggshell membrane. This observation can be attributed to the formation of the air chamber by the eggshell membrane. A poorly formed chamber of deformed eggs could provide an appropriate atmosphere for *Mycoplasma* growth [4]. Furthermore, oviduct infection with mycoplasma can lead to an egg infection, as egg development occurs across the oviduct.

The mutations we observed in the amplified regions of the *vlhA* gene are consistent with previous reports on *vlhA* sequences by El-Gazzar *et al.* [13], demonstrating the variability of this gene. However, previous studies on *Myc. gallisepticum* has reported overexpression of gene regions depending on infection [20,32]. In our field study, we do not know the period of infection in the flocks; nevertheless, we performed our work with the field and ATCC strains under the same conditions, resulting in consistent sequences for the corresponding bioinformatic analysis.

**Fig. 3.**
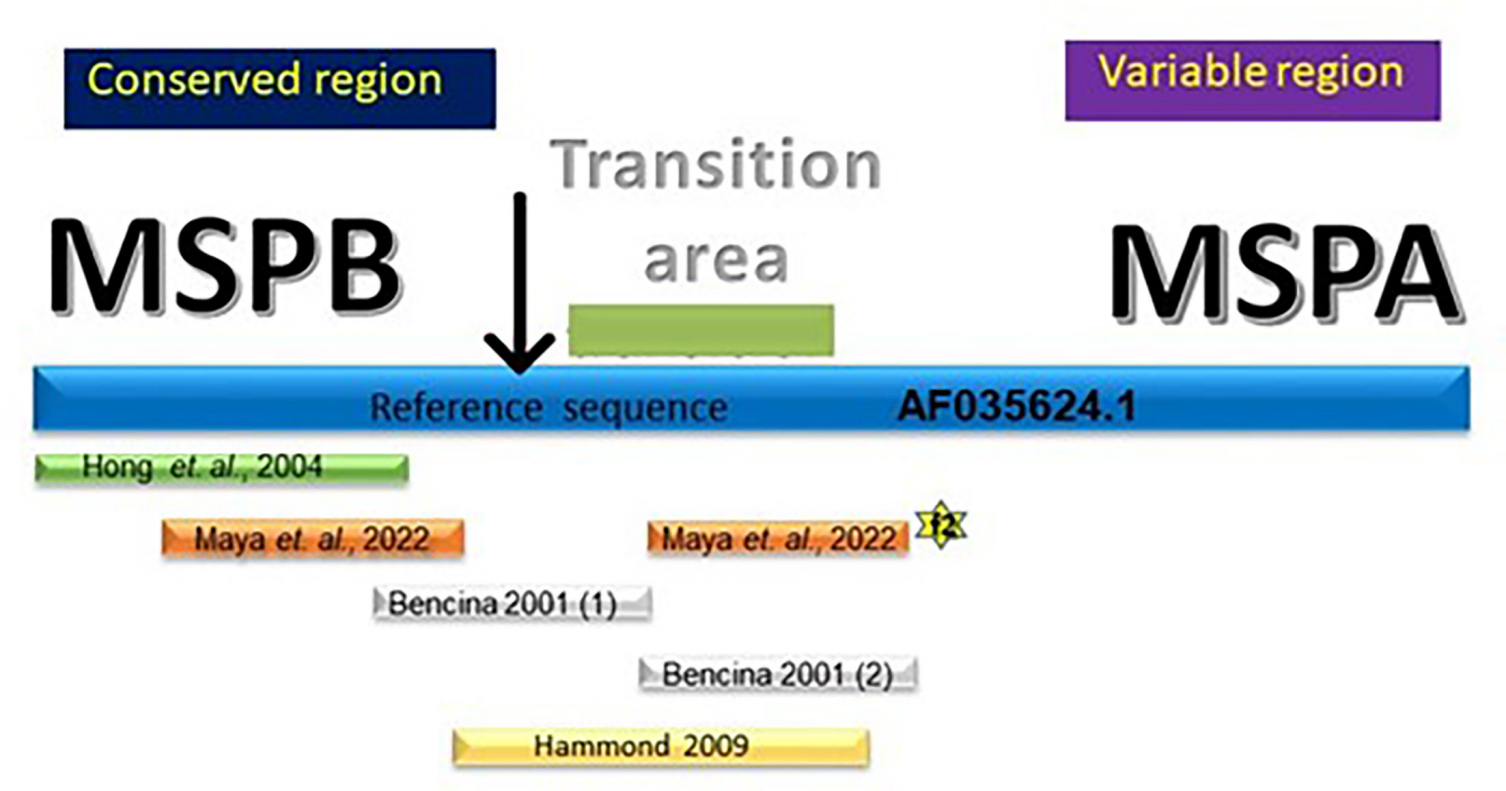
Comparison of the reported primers for the *vlhA* gene (based on the GenBank AF035624.1 reference sequence). The primers reported by different authors, and where they bind to the reference sequence, are presented as coloured bars. Description of the symbols: the black arrow indicates the end of the MSPB segment in the reference sequence. The star indicates MSPA fragment 2. The blue bar represents the AF035624.1 sequence. The green bar represents a transition area between conserved and variable areas. The primers of authors referecence are in fowlling citations [10,11, 33].

The design of primers that amplify the *vlhA* gene (based on AF035624.1 from *Myc. synoviae*) for typing is critical due to mutations that can be found in the sequences of the field strains, especially those designed in the MSPA region [15,33]. This condition could result in false negatives based on PCR because the MSPB region is more stable [13]. The primers recommended by the OMSA for *Myc. synoviae* typing, through amplification of *vlhA* [10], are more stable for amplification in the field strains (Fig. 3). In contrast, the first set of primers reported by Bencina *et al.* [11] is located up to nucleotide 669, whereas the second set of primers reaches nucleotide 1071; therefore, both are between the transition zone and the MSPA region (Fig. 3). Finally, the primers reported by Hammond *et al.* [33] are located up to nucleotide 446, which is in the transition zone between the MSPA and MSPB regions (Fig. 3). In our comparative analysis, the primers reported by Hammond *et al.* [33] were not effective in typing our *Myc. synoviae* field strains, an outcome directly related to the location of the primers within the sequence. Based on this finding, we infer that it is more effective to characterize *Myc. synoviae* by amplifying *vlhA* with primers positioned in the MSPB region (Fig. 3). MSPA fragment 2 – located in the transition zone between the MSPB and MSPA regions – showed >70% homology in the 24 field strains. We have only provided *in silico* analysis of mutations; it is necessary to examine transcription to confirm the results.

The results regarding the conserved sites in both regions are consistent with those reported by May and Brown [34], who found greater homology within the conserved region compared with the variable region. Nevertheless, there were homologous sites within the conserved and transition regions. Similar findings have been reported in studies on haemagglutinin in other micro-organisms. For example, haemagglutinin of the avian influenza virus showed similar genomic regions in strains recovered from ducks in Korea, and genetic analysis revealed stable homologous sites [35].

In other analysed *Mycoplasma* surface proteins, variable regions have been found near the C-terminus of the proteins – for example, the Vsp surface protein of *Mycoplasma bovis* PG45. In another study, the authors determined that the location of the genes has a direct relationship with the capacity for variation of the protein [36]. The protein-coding *vlhA* genes are located in tandem in *Myc. gallisepticum* [37], which could be related to the recombination capacity. In other bacterial genera, the location of the genes can influence the function of proteins. For example, the Hsa protein in *Streptococcus gordonii* has serine-rich repeat sites with sialic acid terminals and *α*2→3 linkages for glycoproteins. This protein facilitates adherence to the host cell, and mutations in the non-repeated regions at the N-terminus directly affect *in vitro* binding to erythrocytes, decreasing its adhesion [38].

**Fig. 4.**
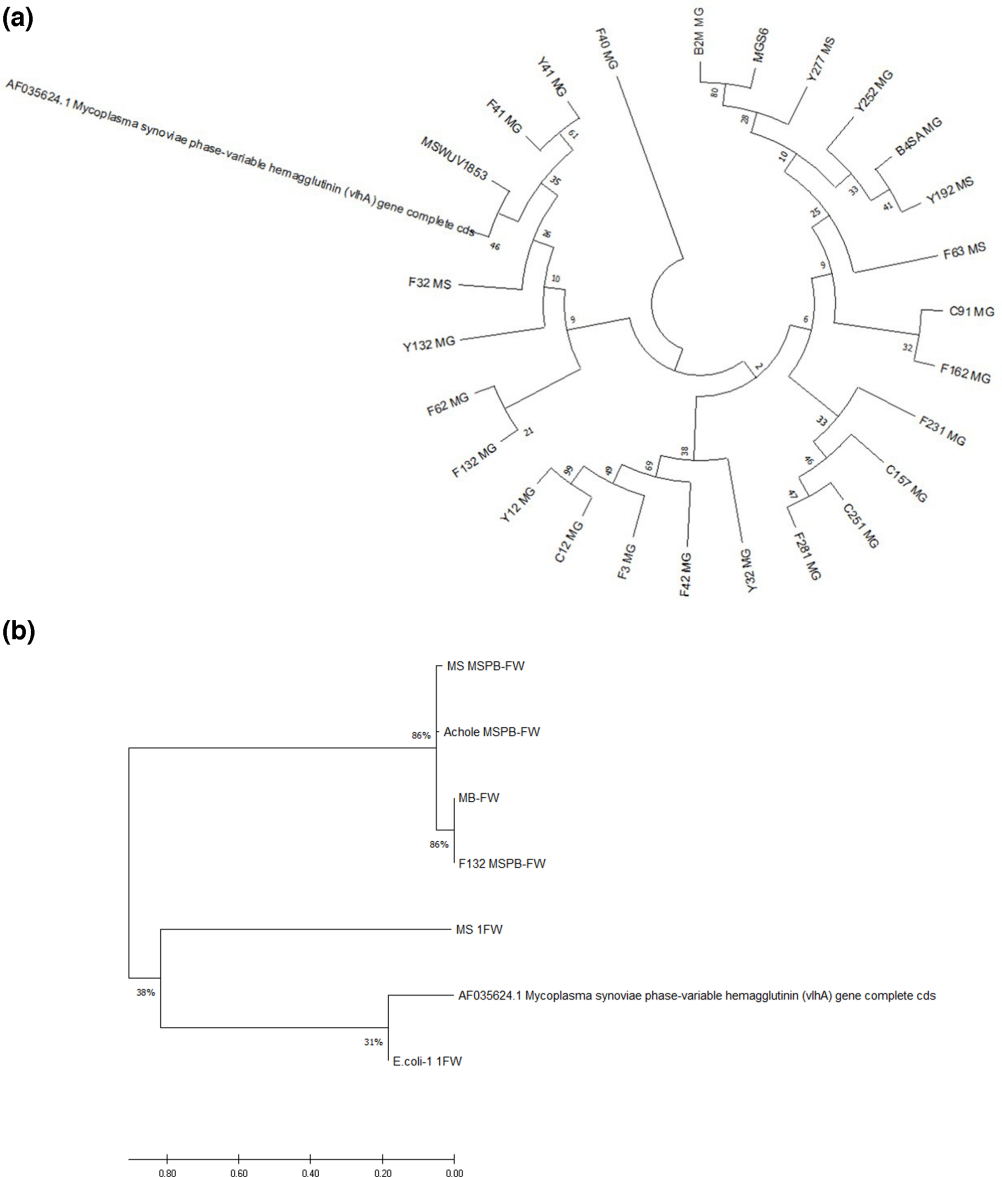
(a) Analysis of the amplified sequences of the MSPB region of *vlhA* field strains (the amplicon was 397 bp). A total of 24 strains were analysed, all of which infect avian species. The percentage of associated taxa clustered together is indicated next to each branch. A heuristic search was conducted automatically by applying the neighbour-joining and Bio NJ algorithms to a matrix of pairwise distances estimated using the Tamura–Nei model. This analysis involved 41 nucleotide sequences, with a total of 2227 positions in the final dataset. Evolutionary analyses were carried out using the mega X software. (b) Analysis of the amplified sequences of the MSPB region of *vlhA* in other bacteria (the amplicon was 397 bp). A total of seven strains were analysed, comprising two samples of *Myc. synoviae* (MS) WUV1853, *E. coli*, F132 field strain, *Myc. bovis* (MB) ATCC, *A. laidlawii* ATCC (Achole MSPB) and the AF035624.1 sequence. The percentage of associated taxa clustered together is indicated next to each branch. A heuristic search was conducted automatically by applying the neighbour-joining and Bio NJ algorithms to a matrix of pairwise distances estimated using the Tamura–Nei model. This analysis involved 7 nucleotide sequences, with a total of 352 positions in the final dataset. Evolutionary analyses were carried out using the mega X software.

**Fig. 5.**
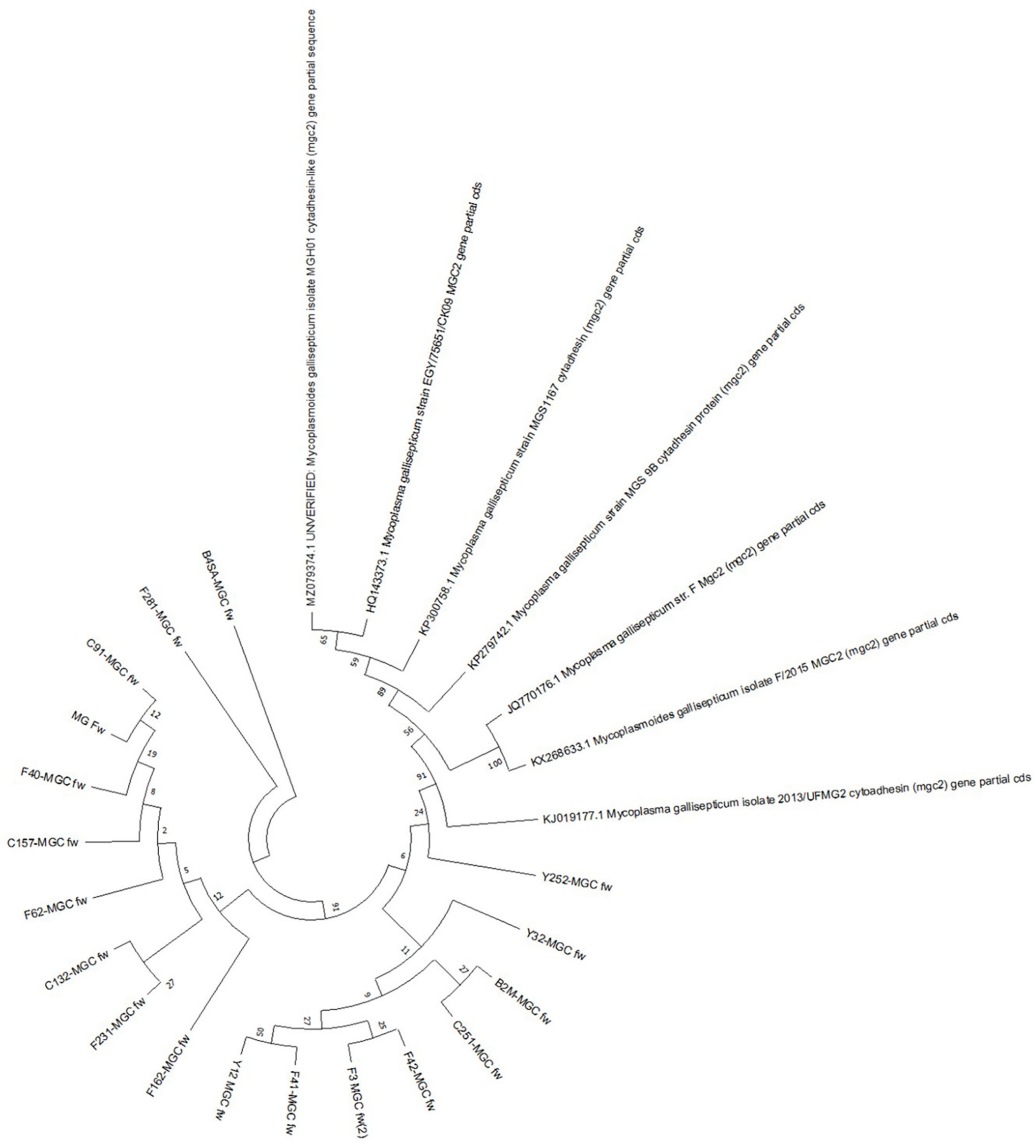
Analysis of the amplified sequences of *mgc2* (the amplicon was 349 bp). The analysis included 17 *Myc. gallisepticum* strains isolated in Mexico, along with sequences retrieved from GenBank. The evolutionary history was inferred using the maximum likelihood method and the Kimura two-parameter model. A total of 25 nucleotide sequences were involved in this analysis, comprising a dataset of 1152 positions. Evolutionary analyses were performed using the mega X software. The GeneBank access for sequences is in Table 4.

**Fig. 6.**
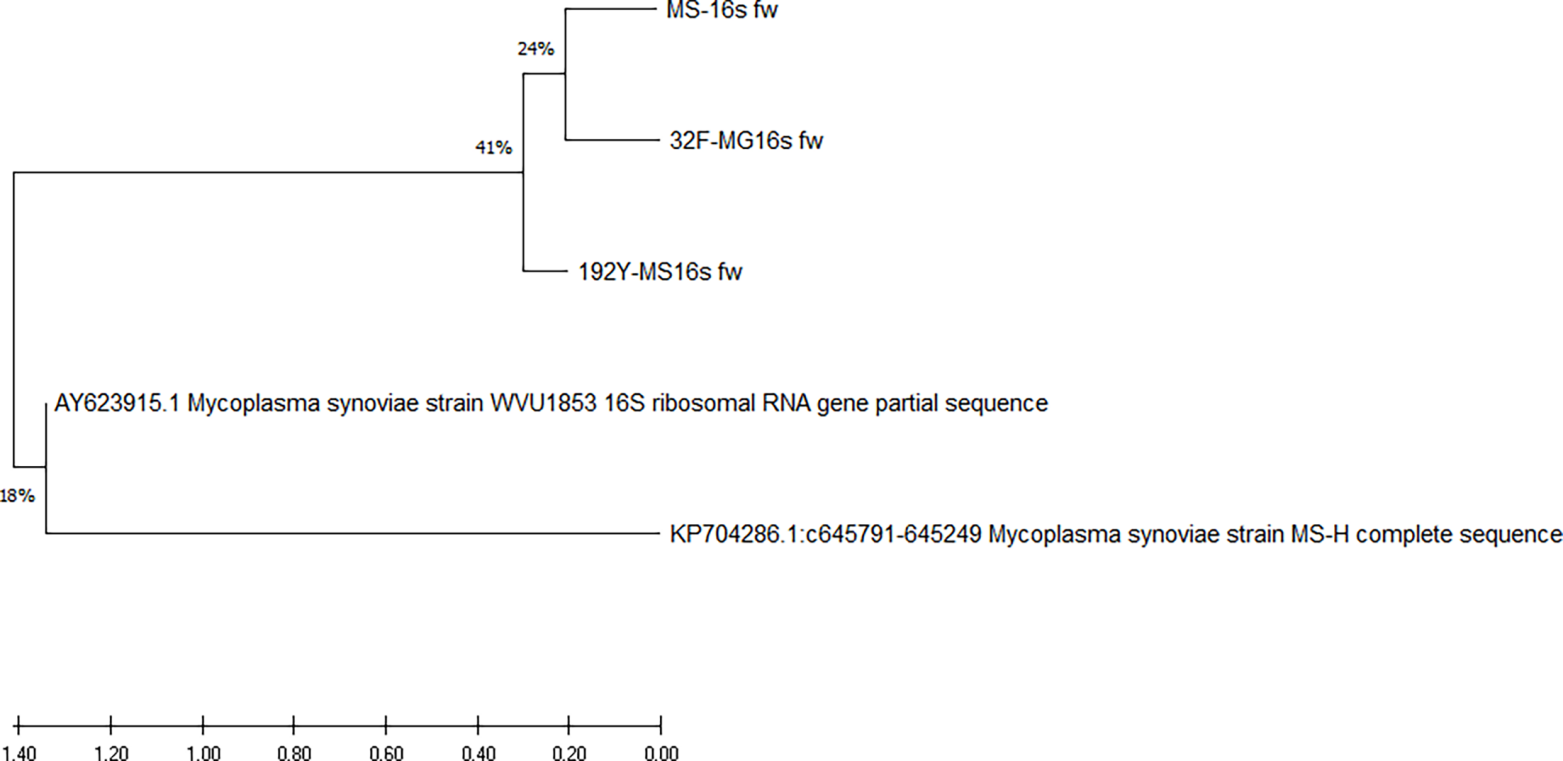
Analysis of the amplified sequences of 16S rRNA: the amplicon was 514 bp for the *Myc. synoviae* strains obtained from this study, and 176 bp for *Myc. gallisepticum* strains reported by Kleven and Bradbury [5]. Additionally, sequences from *Myc. synoviae* reported from Korea and vaccine strains MS-H were retrieved from GenBank. The evolutionary history was inferred using the maximum likelihood method and the Tamura–Nei model. The analysis involved 5 nucleotide sequences, with a total of 544 positions in the final dataset. Evolutionary analyses were conducted using the mega X software. The GeneBank access for sequences are in Table 4.

We confirmed the results of our cluster analysis by performing a comparative evaluation. Eight samples amplified the PCR product from the MSPB region, six of which tested negative for digitonin and, based on PCR, were not *Myc. gallisepticum* or *Myc. synoviae*, suggesting they could be *Acholeplasma* strains. Two samples were identified as *Myc. gallisepticum* but were not isolated, and DNA was only obtained from one of these samples. Given that the phylogenetic trees in Fig. 4a show a close relationship between the strains isolated in Mexico and the sequences reported in GenBank, we performed a cluster analysis based on sequences amplified from *mgc2* (from *Myc. gallisepticum*; Fig. 5) and 16S rRNA (*Myc. synoviae*; Fig. 6). The *mgc2* phylogenetic tree in Fig. 5 shows 17 Mexican strains, and the clade distribution is similar to the phylogenetic tree in Fig. 4a, indicating similarity with other strains and the F strain vaccine, originally isolated from the USA, which is more successful for prophylaxis than other vaccines [5]. The 16S rRNA phylogenetic tree indicates a relation with vaccine strains such as MS-H and a Korean strain, supporting the PCR findings for *Myc. synoviae* WVU1853 (Fig. 6).

In this study, there was positive amplification from *A. laidlawii*, which belongs to the class Mollicutes. *Acholeplasma* spp. have eight major lipoproteins on their surface, and members of Mollicutes can share some proteins. Therefore, it is possible that the N-terminus of a lipoprotein from these taxa could have a similar sequence to the MSPB region, which is a more conserved and stable portion due to its high probability of being maintained in this bacterial phylum [39]. Lipoproteins of Gram-negative bacteria such as *E. coli* have been compared with *A. laidlawii* and have shown similarities in apolipoprotein *N*-acyltransferase (encoded by the *lnt* gene), but *lnt* has not been reported in Mollicutes [39]. Based on the available evidence, lipoproteins from *Acholeplasma* spp., Gram-negative bacteria and some *Mycoplasma* spp. can show a close relationship with epitopes of viral haemagglutinin [39]. However, there are no reports of diseases in domestic animals caused by *Acholeplasma*. It shows a phylogenetic tree with two different samples of *Myc. synoviae* WUV1853 and *E. coli*. We observed a 31% match with AF035624.1, reference sequences and even *S. aureus* ATCC 6538 with lipoproteins (Fig. 4b).

Lipoproteins play a crucial role in the pathogenicity of *Mycoplasma* spp., and understanding the evolution of this genus is important. It is hypothesized that gene transfer among *Mycoplasma* spp. that infect avian species improves bacterial survival [40]. Our finding is that the MSPB region of *vlhA* is similar to our *Myc. synoviae* and *Myc. gallisepticum* field strains, which supports this hypothesis. Paralog genes are utilized in *Myc. synoviae* for modulating on and off processes according to the medium conditions, and recombination genes constantly influence replicating bacteria [41]. The *in vitro* and *in vivo* conditions of the VlhA protein of *Myc. synoviae* and *Myc. gallisepticum* have been studied extensively. This protein is assumed to be important for infection and crucial for diagnosis and target vaccination due to its higher antigen capacity and modulation of the immune system [34]. However, variability in the strain sequences has also been observed. The two homologous regions we characterized should improve the study of haemagglutinin and perhaps even contribute to evolutionary studies.

An ortholog gene is a common ancestral sequence that can be used to measure evolutionary relationships. Ortholog genes are related via speciation (vertical descent), whereas paralog genes are related via duplication [40]. The *vlhA* sequence is necessary for *Myc. synoviae* and *Myc. gallisepticum* to survive, disseminate and infect avian species [41,49]. Paralog genes flanking the *vlhA* gene lead to successful antigenic variability of this protein [41,43, 45, 46]. Based on the two homologous regions found in *vlhA* genes shared by *Mycoplasma* spp. that infect avian species, *A. laidlawii* and *E. coli*, additional studies are necessary to determine whether the sequences are essential for the functions of VlhA and can be considered ortholog sequences [34,45, 49,51]. Similar results based on phylogenetic analysis of the *vlhA* MSPB, *mgc2* and 16S rRNA sequences indicate that the observed homology can be useful for evolutionary analysis.

## Conclusion

In conclusion, the *vlhA* sequence (AF035624.1) exhibits two homologous sequences in the MSPB and MSPA regions, as evidenced by amplification in all 24 field strains (comprising *Myc. synoviae* and *Myc. gallisepticum*). The phylogenetic analysis demonstrated an overlapping distribution of *vlhA* sequences among these two *Mycoplasma* species that infect avian species, which was further supported by the *mgc2* and 16S rRNA phylogenetic trees. The MSPB region shares sequences with *A. laidlawii* and *E. coli*. Therefore, additional investigations are warranted to elucidate the functional significance of both homologous sequences. This would involve verifying whether these sequences represent orthologous genes and assessing their potential functionality in phylogenetic studies and as therapeutic targets. Further exploration in this direction could contribute to a better understanding of the evolutionary relationships and pathogenic mechanisms within *Mycoplasma* species that infect avian species.

## supplementary material

10.1099/acmi.0.000760.v4 Uncited Supplementary Material 1.

## Abbreviations

40×: high power objective lens 40
ATCC: American Type Culture Collection
°C: celsius degrees
kb: millions of bases
µl: microlitre
µm: micrometer
min: minute
ml: millilitre
*Myc*: *Mycoplasma*
pb: pair of bases
PBS: phosphate-buffered saline
PCR: polymerase chain reaction
pH: potential Hidroxin
s: second
Tm: alienation temperature
USA: United States of America
