# Supplementary material for: A comparative in silico analysis of the vlhA gene regions of Mycoplasma gallisepticum and Mycoplasma synoviae isolates from commercial hen farms in Mexico
Source: Access Microbiol. 2025 Feb 21;7(2):000760.v4. doi: 10.1099/acmi.0.000760.v4 (PMC11845793; doi:10.1099/acmi.0.000760.v4)
Supplement: Uncited Supplementary Material 1. [file acmi-7-00760-s001.pdf]

>AF035624.1 Mycoplasma synoviae phase-variable hemagglutinin (vlhA) gene complete cds

AATTTAGAGCTCAATTTGTAACAAATTCAATAAACGGAGTTACTATAACTAAAGTTCAAAACACAAAAGAGCTA
AGACCTGGAACTTTAGACGATTTACTAAAAAATAGGAACAACGTATTCTTACAGCAAATCCAAGGAGATACTG
AAGCAGTGTATTTTGCAGTAACTGCAATAGCAAGCAATAGTTGATTAAATACCTTCTTAATTAGAATACCTTTAA
CTAAATTTGTTAAACCTTTAACAGAATTCAGACC-TACAACTCCTACAAGCC-
CTTCTAGCGATACTCAACAACAAGGAACTGCTCAAACTCAAAGTAATCAAGGTTAATAATAACAAAACCAAAA
ACAT

>MS_MSPB-FW (sample# 1 *Myc. synoviae* WUV1853)

--------------------GGGAGTCGAGACGCTGAGCCG-
AGATGGTAATTTAGAGCTCAATTTGTAAACAAATTCTTGTCGGTGTTTTCTTCCCC-
CTTCTTTATGATCTGTTGAATCATATTCAGGTATCTTGATCTTGCTTAAAACGTGTTTCACTTGATCTTGGACAGG
GTCAGCAGAAGCTGAACCCAGAGATCGTTCTATTAAAGAAAATGAAGAACTTTGCACCTAC-
GAGCGTGTAGCACGCCCGATGCGTTCTCTATACTTC-
GTATCAACAGTTGCTATTTTCTTCGCAAACTTAGGACTTGCAGAAGCTGAAGCC-AGAGA----------------

>E.coli-1 _1FW (sample *E. coli* CVET0058 strain)

TACCTTCATAATTCGAATACCTTTAAATAATTTTGGTAAACCTTTAACTGAATTCAGCTC-TACAACTCCTACAA-
CC-CTTCTAGCGATAC--AGCTATCTGG--CTACTCTGGC--------------------------------------

>Achole_MSPB-FW (sample *Acholeplasma laidlawii* PG8)

--------------------ACTAGTCGAGATGCTGAGCCTCAGATGGTAATTTAGAGCTCAATTTGTAA-
CAAATTCTTGTCGGTGTTTTCTTCCCC-
CTTCTTTATTATCTGTTGAATCAGATTCAGGTATCTTGATCTTGCTTAAAACGTGTTTCACTTGATCTTGGACAGG
GTCAGCAGAAGCTGAAGCCAGAGATCGTTCTATTAAAGAAAATGAAGAACTTTGCACCTAC-
GAGCGTGTAGCACGCCCGATGCGTTCTCTATACTTC-
GTATCAACAGTTGCTATTTTCTTCGCAAACTTAGGACTTGCAGAAGCTGAAGCC-AGAG-----------------

>MB-FW (sample *Mycoplasma bovis* strain PG45)

--------------------ACTAGTCGAGACGCTGAGCCAGAGATGACAAT----------ACTTGCTCGCCA--
CATTGTCGGTGTTTTCTTCCCAACTTCTTTATTATCTGTTGAATCATATTCTGGTATCTTGATCTTGCTTAGAACGT
GTTTCACTTGATCTTGGACAGGGTCAGCAGAAGCTGAAGCCAGAGATCGTTCTATTAAAGAAAATGAAGAAC
TTTGCACCTACAGAGCGTGTAGCACGCCCGATGCGTTCTCTATACTTC-
GTATCAACAGTTGCTATTTTCTTCGCAAACTTAGGACTTGCAGAAGCTGAAGCCCAGAGA----------------

>F132_MSPB-FW (sample field strain F132 *Mycoplasma gallisepticum*)

--------------------ACTAGTCGAGACGCTGAGCCAGAGATGACAAT----------ACTTGCTCGCCA--
CATTGTCGGTGTTTTCTTCCCAACTTCTTTATTATCTGTTGAATCATATTCTGGTATCTTGATCTTGCTTAGAACGT
GTTTCACTTGATCTTGGACAGGGTCAGCAGAAGCTGAAGCCAGAGATCGTTCTATTAAAGAAAATGAAGAAC
TTTGCACCTACAGAGCGTGTAGCACGCCCGATGCGTTCTCTATACTTC-
GTATCAACAGTTGCTATTTTCTTCGCAAACTTAGGACTTGCAGAAGCTGAAGCCCAGAGA----------------

>MS_1FW (sample #2 *Myc. synoviae* WUV1853)

GTCCATCTGGAACTCTGGCTTCAGCATCTGGCACTCTGGCTTCAGCTTCTGGCTCTCTGGCTTCAGCTATCTGG
CTCTCTGGCTTCAGCTTCTGGCTCTCTGGCTTCAGCTTCTGGCTCTCTGGCTT-CAGCTTCTG---------------------
----
